# Peritoneal Equilibration Test in Costa Rica: Discrepancies from Other Populations

**DOI:** 10.1155/2014/326163

**Published:** 2014-01-02

**Authors:** Marta Avellan-Boza, Fabio Hernández, Allan Ramos-Esquivel

**Affiliations:** ^1^Department of Nephrology, Hospital San Juan de Dios, 11501-2060 San José, Costa Rica; ^2^Department of Pharmacology, University of Costa Rica, 11501-2060 San José, Costa Rica

## Abstract

*Objective*. Continuous Ambulatory Peritoneal Dialysis (CAPD) is a kidney replacement therapy that has been recently incorporated in developing countries. We aim to establish our reference values, to compare them with the original and the Mexican population, and to associate some variables with the type of peritoneal transport. *Methods*. Thirty peritoneal equilibration tests (PET) were performed. The ratio for D/P creatinine and the D/D_0_ ratio for glucose were calculated and compared to reference values. We conducted a retrospective analysis to correlate peritoneal transporters with some predictive variables. *Results*. D/P creatinine ratio at 2 hours, D/D_0_ glucose ratio at 4 hours, and net ultrafiltrate volume (nUFV) were significantly different from those reported by Twardowski et al. The results documented in the Mexican population only coincide with our results for the D/P creatinine ratio at 4 hours. Any of the studied variables were associated with a specific type of peritoneal transport. *Conclusions*. Peritoneal permeability among Costa Rican CAPD patients is different from the original population described by Twardowski et al. and from other Latin-American population. This supports the theory that ethnical differences could be responsible for such variations and they validate our statement that each region should possess value references of their own.

## 1. Introduction

In Costa Rica, continuous ambulatory peritoneal dialysis (CAPD) was introduced in the late eighties, initially with few patients as a short-term renal replacement therapy. Currently, social security covers around one hundred fifty adult patients (2/3 of which are men) distributed in four main hospitals around the country.

Patients undergoing CAPD exhibit a wide variability in the peritoneal transport of solutes and fluids. Measuring the characteristics of peritoneal membrane transport is important for the characterization of the functional state as for the prescription of the adequate dose of dialysis therapy in an individual fashion [1, 2]. The peritoneal equilibration test (PET) is the most widely used method to characterize and classify the peritoneal transport of patients on dialysis, and their methodology was standardized by Twardowski et al. [2, 3] more than two decades ago. The PET characterizes the peritoneal membrane transport properties by determining the ratio of the creatinine concentration in the dialysate to that in the plasma after a 4 h dwell (D/Pc) and has been shown to vary considerably among individuals. However, even if the results are highly consistent in the same individual, great variability has been seen among patients [4]. The PET is an easy and inexpensive test and it provides valuable information about peritoneal clearance of solutes and ultrafiltration. Peritoneal transport type classification is broadly recognized not only as aid for prescription but also as a prognostic index since previous analyses have indicated that a higher rate of peritoneal membrane solute transport is associated with increased mortality in peritoneal dialysis patients [5].

In our country an adequate characterization of the type of peritoneal transport has not been done yet. Our population presents marked ethnic differences as compared with those for which the test was described. In previous studies significant differences have been found in reference values for D/D_0_ glucose and D/P creatinine for different populations using the same standardized PET [6–8].

For these reasons, we aim to establish the reference values for the Costa Rican population and to determine the influence of different clinical factors on the type of peritoneal transport.

## 2. Subjects and Methods

The standardized Twardowski et al. [3] PET was performed in thirty consecutive adult outpatients belonging to the CAPD program at San Juan de Dios Hospital, San José, Costa Rica, in the period between August and October of 2012. Patients that presented with peritonitis in the last 4 weeks or that had less than 4 weeks since placement of the catheter were excluded.

Following the night exchange and 8–12 hours of dwell time, the totality of the abdominal cavity was drained and new exchange was introduced with 2 liters of glucose 4.25% peridial solution this was performed in the supine position and mobilizing the patient every 2 minutes to insure an adequate mixture of the dialysate solution in the abdominal cavity. Shortly after, dialysis solution samples and blood samples were taken at 0, 2, and 4 hours. After 4 hours, the dialysate solution was drained and its volume was quantified. Glucose, creatinine, C reactive protein, and albumin were measured in each sample, using conventional techniques with a colorimetric assay. There was no need to use are a correction factor for creatinine in the peritoneal fluid, because it was proven that its quantification with the equipment used is zero, in spite of the high glucose concentrations in a new peridial solution bag [9].

Using our own values for D/P creatinine, a patient was classified as a high transporter if his D/P is 1 standard deviation (SD) above the mean (>0.78); as high-average if his D/P is between the mean and +1 SD (0.66–0.78); as low-average if his D/P is between the mean −1 DS (0.54–0.65); and it was classified as low transporter if his D/P is found below −1 DS (<0.53) [1, 2]. The study protocol and consent form were approved by the local institutional review board and ethics committee. All subjects or an appropriate surrogate provided written informed consent before any study-related procedure was completed.

A sample size of thirty patients was calculated with the objective of determining the proportion of average transporters (high and low), with an 18% of precision. We took as reference an expected proportion of average transporters of 68%, as suggested by previous studies [4].

The data is presented as mean ± SD or percentages. The comparisons between our results and those reported by Twardowski et al. [3] and by Cueto-Manzano et al. [4] were made through the Student's *t* test for independent samples. The comparisons between the type of transporters according to the D/P creatinine and D/D_0_ glucose were made by the ANOVA test, square chi test, or by the exact Fisher test according to the type of variable. We decided to measure only the D/P creatinine and D/D_0_ glucose rates because these are the most useful indexes to predict patient responses to peritoneal dialysis according to Twadorsky et al. [3].

A *P* value ofless than 0.05 was considered as significant. All statistical analyses were carried out using SPSS 20.0 for Windows (Chicago, Illinois).

## 3. Results

The characteristics of the population studied are represented in Table 1. In average, the cohort was mainly composed by men whose therapy time varied between 8.6 and 154 weeks, with a mode of 52 weeks. None of these variables was statistically related with any type of peritoneal transport, neither with the presence of peritonitis nor diabetes mellitus (Table 2).

The comparisons of the PET values obtained by D/P creatinine and by D/D_0_ glucose at 2 and 4 hours in our population and those obtained in the original population by Twardowski et al. [3] and by Cueto et al. [4] are shown in Table 3. The results of D/D_0_ glucose at 4 hours, of D/P creatinine at 2 hours and the ultrafiltrate volume are different in our population than those found in the population studied by Twardowski and colleagues. The results documented in the Mexican population only coincide with our results for the D/P creatinine values obtained at 4 hours.

## 4. Discussion

In Latin America very few studies have been made to validate the PET in this region. PET is a feasible technique used to tailor the CAPD prescription and a very important tool to predict poor outcomes. Since much variability has been observed in the PET results, it is recommended to perform this test early in treatment in each CAPD Unit [10].

If our sample were categorized according to the reference values provided by Twadorski et al. [3], the classification of our cohort would be changed. The population distribution, according to our values, would vary with respect to those found by Twardowski, since, according to our curves, a 16.6% are low transporters, 36.6% are low-average transporters, 36.6% are high-average transporters, and around 10% are high transporters. If they were classified in accordance with the original population the distribution would be 10% low transporters, 46.7% low-average transporters, and 43.3% high-average transporters, hence, no patient would be classified as a high transporter.

Besides, in comparison with the Mexican cohort, our sample exhibited a lower creatinine D/P ratio at 0 hours. This can be the result of heterogeneous residual dialysate volumes. However, neither the Mexican study nor our research determined this volume and proper conclusion could not be drawn. Similarly, glucose D/D_0_ ratios at 2 and 4 hours were lower in our cohort than in the Mexican and original population. This result may be due to high dialysate glucose absorption as well as a consequence of eventual errors in nursing procedures or laboratory measurements.

We must highlight that the composition of each compared population was different. Sixty-three percent of our sample was diabetic and one third of our patients had a history of peritonitis. Both of these variables were significantly different in our sample and can also explain our findings. Several studies, with conflicting results, have revealed histopathological alterations in the peritoneal membrane of diabetic patients leading to increased glucose absorption [3, 4]. Furthermore, patients with past peritonitis also exhibit high values of monocyte chemoattractant protein-1 (MCP-1) resulting in increased peritoneal membrane permeability [11].

It is remarkable that the ultrafiltrate volume is one of the variables that differ in a greater magnitude, both in the original population and in the Mexican cohort, since the volume variable was larger in our sample. This is not in concordance with our findings of D/D_0_ for glucose, which is significantly smaller at 4 hours and indicates larger glucose absorption from the dialysis solution. It suggests that other nonosmotic stimuli can explain how the ultrafiltrate volume independently varies from the glucose concentration of the dialysate. In animal models, it has been suggested that 50% of the transperitoneal water flow occurs through ultrasmall pores, known as aquaporins 1 whose expression is not completely related with osmotic stimuli [1, 12].

It is important to note that in comparison with the original and Mexican population we used a 4.25% dextrose solution instead of the 2.5% dextrose exchange. Although previous findings have determined that D/D_0_ glucose and D/P creatinine are similar with the use of either 4.25% or 2.5% solution, these studies have also shown that using a 4.25% dextrose solution produce higher drained volumes than the 2.5% dextrose exchange [13]. Hence, disparities in drained volumes can also be explained as a consequence of different dialysis solutions.

Although differences in body mass composition (due to the majority of men in our sample or secondary to ethnic differences) can explain our results, the actual evidence regarding this issue is inconclusive. Previous investigations have shown the relationship between large body surface area and high D/P creatinine [14], while other studies have reported a null effect [15]. Besides, the high proportion of men patients in our cohort reflects the Costa Rican peritoneal dialysis population, with almost two-thirds of patients being male.

Although we did not determine any genetic factor that explains the variability among the three cohorts, several gene polymorphisms in the vascular endothelial growth factor (VEGF) gene, endothelial nitric oxide synthase gene (ENOS), and interleukin 6 (IL-6) gene have been correlated with the interpatient variability in PET results [16]. Further studies must test this hypothesis in order to identify genetic variants that explain these differences among populations.

In this study there were no significant differences between age, gender, diabetes mellitus, serum albumin, C reactive protein, and the type of peritoneal transport, even when previous studies have revealed a higher proportion of men, diabetic patients, and low serum albumin concentration in the high transporter group [1, 17]. These conflicting results can be the consequence of the different threshold to define high average transporters in our sample, as well as an under representativeness of these subgroups.

## 5. Conclusions

Studies performed in other latitudes show different reference ranges for the classification of the types of peritoneal transport. The PET results of this study differ from the values described originally by Twardowski et al. and from other Latin American population. As a consequence of these discrepancies, we consider that CAPD prescription in our country should be made based on the presented reference values.

Our findings support the theory that different ethnical factors can be responsible for such variations and it validates the affirmation that each region should possess its own reference values.

## Figures and Tables

**Table 1:** Characteristics of the population studied in the three compared groups.

Variable	Twardowski cohort [3] (*n* = 86)	Mexican cohort [4] (*n* = 86)	Costa Rican cohort (*n* = 30)
Age (years)	NR	45.6 ± 16.0	60.23 ± 11.55
Male gender (%)	NR	41 (47.7)	25 (83.33)^‡^
Diabetes mellitus (%)	18 (20.9)	35 (40.7)	19 (63.33)^∗‡^
Therapy time (weeks)	21.22 ± 12.2	83.2 ± 172	39.55 ± 34.7*
Peritonitis during treatment (%)	0	2 (2.3)	9 (30)^‡^
Albumin (g/dL)	NR	3.23 ± 0.7	2.97 ± 0.5
C reactive protein (mg/dL)	NR	0.98 ± 0.2	0.97 ± 1.2

NR: not reported.

*Value of *P* < 0.05 for the comparison between the Twardowski and the Costa Rican cohorts.

^‡^Value of *P* < 0.01 for the comparison between the Mexican and the Costa Rican cohorts.

**Table 2:** Peritoneal transportation according to the clinical characteristics of the population under study.

Variable	Type of transporter				*P* value
	Low *n* = 5	Low-average *n* = 11	High-average *n* = 11	High *n* = 3	
Age (years)	62.2 ± 16.9	62.6 ± 7.7	55.3 ± 12.6	66.3 ± 5.7	0.34
Male gender (%)	5 (20)	9 (36)	8 (32)	3 (12)	0.47
Diabetes mellitus (%)	4 (21.1)	8 (42.1)	6 (31.6)	1 (5.3)	0.47
Peritonitis (%)	1 (11.1)	4 (44.4)	3 (33.3)	1 (11.1)	0.92
Dialysed volume (L)	3.11 ± 0.2	2.83 ± 0.3	3.10 ± 0.3	2.90 ± 0.6	0.19
Therapy time (weeks)	27.5 ± 18.6	44.6 ± 45.6	30.5 ± 18.5	74.5 ± 42.3	0.20
Albumin (g/dL)	3.12 ± 0.1	3.01 ± 0.5	2.87 ± 0.7	3.00 ± 0.4	0.84
C reactive protein (mg/dL)	0.42 ± 0.1	0.95 ± 1.4	1.27 ± 1.2	0.81 ± 1.2	0.62

**Table 3:** Comparison between the ratio of solutes and dialysis volume drained in the original, the Mexican, and the Costa Rican population.

	Twardowski et al. [3] (*n*)	Mexican cohort [4] (*n) *	Costa Rican cohort (*n*)
D/P creatinine 0 h	0.07 ± 0.05 (86)	0.12 ± 0.06 (86)	0.05 ± 0.04 (30)^‡^
D/P creatinine 2 h	0.48 ± 0.14 (101)	0.48 ± 0.10 (86)	0.36 ± 0.09 (30)^∗‡^
D/P creatinine 4 h	0.65 ± 0.16 (101)	0.68 ± 0.12 (86)	0.66 ± 0.12 (30)
D/D_0_ glucose 2 h	0.55 ± 0.11 (86)	0.59 ± 0.08 (86)	0.53 ± 0.13 (30)^‡^
D/D_0_ glucose 4 h	0.38 ± 0.11 (85)	0.39 ± 0.09 (86)	0.29 ± 0.05 (30)^∗‡^
Drained volume (L)	2.37 ± 0.28 (94)	2.46 ± 0.18 (86)	2.99 ± 0.33 (30)^∗‡^

*Value of *P* < 0.05 for the comparison between the Twardowski and the Costa Rican cohorts.

^‡^Value of *P* < 0.01 for the comparison between the Mexican and the Costa Rican cohorts.

## Abbreviations

CAPD: Continuous ambulatory peritoneal dialysis
PET: Peritoneal equilibration test.
